# MoCul4 assists MoKmt1 to catalyze H3K9 methylation and regulates the growth, development, and pathogenicity in *Magnaporthe oryzae*

**DOI:** 10.1080/21505594.2026.2715212

**Published:** 2026-09-11

**Authors:** Pengyun Huang, Chaohao Xu, Jing Wang, Yunran Zhang, Huijuan Cao, Jiale Wang, Haiping Qiu, Xiao-Hong Liu, Jiaoyu Wang, Fu-Cheng Lin, Jianping Lu

**Affiliations:** a School of Medicine, Linyi University, Linyi, Shandong, China; b State Key Laboratory for Quality and Safety of Agro-Products, Key Laboratory of Agricultural Microbiome (MARA), Key Laboratory of Agriculture Microbiome of Zhejiang Province, Key Laboratory of Biotechnology in Plant Protection of MARA, Institute of Plant Protection and Microbiology, Zhejiang Academy of Agricultural Sciences, Hangzhou, China; c Xianghu Laboratory, Hangzhou, Zhejiang, China; d Institute of Plant Protection, Jiangsu Academy of Agricultural Sciences, Nanjing, China; e Institute of Biotechnology, Zhejiang University, Hangzhou, China; f College of Life Sciences, Zhejiang University, Hangzhou, China

**Keywords:** *Magnaporthe oryzae*, H3K9 methylation, histone methyltransferase, Cullin4, pathogenicity, gene expression profiles

## Abstract

Rice blast, caused by the filamentous fungus *Magnaporthe oryzae*, is a serious disease of rice. In this study, by using homologous recombination, gene knockout mutants of Δ*Mokmt1* (a histone methyltransferase) and Δ*Mocul4* (Cullin4 subunit of an E3 ubiquitin ligase) were generated. Phenotypic analysis revealed that both MoKmt1 and MoCul4 are essential for fungal growth, development, and pathogenicity. Compared to the wild-type strain, the Δ*Mokmt1* mutant exhibited a slower growth rate (11.6% reduction) and significantly decreased virulence. Western blot analysis confirmed a significant decrease of H3K9 trimethylation (H3K9me3) levels in Δ*Mokmt1*. These results illustrate MoKmt1 is the key enzyme catalyzing H3K9me3 in *M. oryzae*, which is critical for fungal growth, development, and pathogenicity. Notably, the Δ*Mocul4* mutant displayed similar but more severe developmental defects than Δ*Mokmt1*, including a 32.8% reduction in growth rate, 90.4% decrease in conidiation, and a significant reduction in lesion area. Both MoCul4 and MoKmt1 were localized in the nucleus. Like Δ*Mokmt1*, H3K9me3 levels in Δ*Mocul4* were nearly abolished, suggesting that MoCul4 modulates the methyltransferase activity of MoKmt1. RNA-seq analysis further revealed that 1,033 out of 2,910 differentially expressed genes (35.5%) were co-regulated by both MoKmt1 and MoCul4. The expression level of *MoMAT1-1* and *MoMAT A1-1*, located in heterochromatin, was upregulated in both mutants, indicating compromised integrity of heterochromatin. This study demonstrates that MoCul4, functioning in the same pathway as MoKmt1 in *M. oryzae*, orchestrates the MoKmt1-mediated H3K9me3 epigenetic regulatory network, thereby providing a theoretical foundation for developing novel fungicides targeting epigenetic regulation.

## Introduction

*Magnaporthe oryzae*, a classic model pathogen for studying fungal-plant interactions, exhibits a well-defined infection cycle comprising several key biological processes, such as hyphal growth, conidiation, appressorium differentiation, and host invasion [1,2]. The temporal transition between these developmental stages is precisely regulated by multi-layered regulatory networks [3]. Extensive studies have elucidated the gene expression regulatory mechanisms in *M. oryzae*, including DNA methylation [4,5], transcription factors [6], non-coding RNAs [7], and histone post-translational modifications (PTMs) [4]. DNA methylation guides transcriptionally inactive heterochromatin formation through altering DNA-protein binding and blocking transcription factor access, thereby governing cell differentiation or gene silencing [4,5]. Histone epigenetic modification levels were dynamically balanced by the activities of modifying and de-modifying enzymes in response to environmental signals, thereby modulating chromatin conformation and genome DNA accessibility [8]. Specific histone PTMs recruit transcription factors or chromatin-binding proteins to establish spatiotemporal expression patterns of target genes [9]. In addition to DNA methylation, histone PTMs are highly diverse, primarily including acetylation, ubiquitination, and methylation. Among these, methylation can occur on multiple lysine (K) residues, and its functional outcome is site-dependent, playing different roles in transcriptional regulation. In general, certain methylation marks are associated with active euchromatin, while others serve as hallmarks of repressive heterochromatin. In *Saccharomyces cerevisiae*, the functions of H3K4 and H3K36 methylation, both classic euchromatic marks, have been studied. H3K4 trimethylation (H3K4me3) is predominantly enriched at the promoter regions of actively transcribed genes, where it functions as a transcriptional activator. This modification is catalyzed by Set1, which was the first H3K4 methyltransferase to be identified and serves as the core catalytic subunit of the COMPASS complex [10]. H3K36me3 is deposited across transcription elongation regions by Set2 coupled with RNA polymerase II, thereby facilitating transcriptional elongation [11]. In *Schizosaccharomyces pombe*, H3K9me2/3 serves as a hallmark of constitutive heterochromatin and is catalyzed by the Clr4 methyltransferase. Then the HP1 homolog Swi6 mediates chromatin compaction to achieve stable transcriptional repression [12]. In *Neurospora crassa*, H3K27me3 is catalyzed by the PRC2 complex and serves as a hallmark of facultative heterochromatin, which also represses transcription. However, the underlying mechanism differs from that of the H3K9me3-mediated silencing pathway [13,14]. Chromatin remodeling mediated by histone epigenetic modifications, cooperating with transcription factor networks, has emerged as a critical approach in deciphering the molecular pathogenesis of *M. oryzae*.

Significant progress has been made in elucidating the roles of histone PTMs in gene expression regulation, development, and pathogenicity of *M. oryzae*. The histone deacetylase MoRpd3, whose knockout is lethal, has been shown to suppress conidial cell death via TOR-mediated signaling, thereby negatively regulating appressorium maturation and virulence of *M. oryzae* [15,16]. The Tig1 histone deacetylase complex (core components: MoTig1, MoSet3, MoSnt1, MoHos2) not only regulates sporulation but also participates in activating pathways that resist plant defense responses to promote post-invasion hyphal expansion of *M. oryzae* [17]. During appressorium development, MoSnt2 activates the TOR pathway to induce H3 deacetylation, upregulating autophagy-related genes (*MoATG6*, *MoATG15*, *MoATG16* and *MoATG22*) and facilitating appressorium formation of *M. oryzae* [18]. The histone H3K4 methyltransferase MoSet1 mediates H3K4 methylation. The Δ*Moset1* mutant exhibits reduced H3K4me1/2/3 levels, growth rate, conidiation and pathogenicity [19]. Loss of the H3K4me3 demethylase *MoJMJ1* leads to aberrant H3K4me3 hypermethylation, impairing fungal growth, sporulation, appressorium development, and virulence of *M. oryzae* [20]. H3K27me3 and H3K9me3 are hallmark modifications of facultative and constitutive heterochromatin, respectively, and genes within these regions are transcriptionally repressed [21]. Studies have demonstrated that the Polycomb Repressive Complex 2 (PRC2), comprising conserved subunits MoKmt6 (H3K27me3 methyltransferase), MoSuz12, MoEed, and MoP55, catalyzes H3K27 trimethylation to regulate gene silencing, hyphal growth, conidiation, and infection processes of *M. oryzae* [22,23]. Notably, the MoSin3 histone deacetylase complex is responsible for maintaining the epigenetic occupancy of H3K27me3 to ensure stable repression of target genes [23]. The H3K9 methylation is catalyzed by MoKmt1 in *M. oryzae*, which is the homolog of Clr4 in *S. pombe*, and the knockout of *MoKMT1* reduced H3K9me3 levels and pathogenicity [19]. These epigenetic modifications do not function in isolation but are tightly interconnected. A key finding was that the loss of H3K9me3 specifically triggered the reduction of H3K27me3 levels within the K4-fHC subcompartment, which was enriched for infection-related genes, and eventually destabilized the expression of pathogenicity genes [21]. Although the role of MoKmt1 in pathogenicity has been established, its precise catalytic mechanism and potential requirement for accessory proteins or cofactors remain elusive.

In the epigenetic regulatory systems of eukaryotes, the methylation mechanism at the H3K9 site exhibits remarkable evolutionary conservation. The Cullin4 (Cul4)-scaffolded ubiquitin ligase complex plays a central role in H3K9 methylation and heterochromatin formation. In *Homo sapiens*, the nuclear-localized Cul4B protein acts as a molecular scaffold, forming a functional complex with the H3K9-specific methyltransferase Suv39H1 and cooperating with DNA methyltransferase 3A (Dnmt3A) to catalyze the co-deposition of H3K9me3 and DNA methylation, thereby establishing stable heterochromatin structures and mediating gene silencing [24]. Additionally, Cul4B enhances transcriptional repression by facilitating H3K27me3 deposition at specific loci through its interaction with PRC2 [25]. Depletion of *CUL4B* leads to significant reductions in H3K9me3 and H3K27me3 levels and aberrant activation of heterochromatic genes [26]. *CUL4A* and *CUL4B* overexpression is implicated in tumor progression, metastasis, and a poorer survival rate for patients with lung, colorectal, cervical, esophageal, and breast cancers [27,28]. In *Schizosaccharomyces pombe*, Cul4 interacts with the H3K9 methyltransferase Clr4, Pip1/Rbx1, and Rik1 to form the Clr4 methyltransferase complex (CLRC). Cul4 assists the enzymatic activity of Clr4 and promotes the recruitment of the heterochromatin protein Swi6 to maintain chromatin structure, thereby affecting heterochromatin assembly [29]. Studies in *Neurospora crassa* also reveal that the function of the H3K9 methyltransferase Dim-5 strictly depends on the chaperone function of Cul4 [30]. Beyond facilitating H3K9 methylation establishment, Cul4 exhibits diverse roles across species. In *Caenorhabditis elegans*, Cul-4 maintains genome stability by restraining DNA replication licensing [31] and regulates chromatin segregation during germ cell meiosis [32]. In *Drosophila melanogaster*, Cul-4 maintains genomic integrity by promoting the degradation of cell cycle progression factors and pro-survival regulators [33], and plays a critical role in retinal differentiation [34]. In *M. oryzae*, MoCul4 interacts with MoCand2, which acts as a suppressor of ubiquitination, and the Δ*Mocul4* mutant exhibits enhanced autophagic flux [35], yet the roles of MoCul4 in histone methylation and regulation of gene expression remain unclear. Meanwhile, the study of Cul4 in other phytopathogenic fungi remains largely unexplored. The H3K9 methyltransferases (Suv39H1/Clr4/Dim-5) and the Cullin4 subunit of an E3 ubiquitin ligase exhibit conserved protein sequences and functions across species [24,29,30], suggesting that the *M. oryzae* H3K9 methyltransferase MoKmt1 (the homolog of Suv39H1/Clr4/Dim-5) may recruit MoCul4 (the homolog of the Cullin4 subunit of an E3 ubiquitin ligase) through a similar mechanism, but their precise interaction remains to be elucidated.

In this study, MoKmt1 and MoCul4 were identified in *M. oryzae* as the homologs of the *S. pombe* H3K9 methyltransferase Clr4 and Cul4, respectively. Using homologous recombination, we generated deletion mutants Δ*Mokmt1* and Δ*Mocul4*. Our findings demonstrate that MoCul4, which is essential for the proper function of MoKmt1 in H3K9me3 establishment, regulates fungal growth, sporulation, pathogenicity, H3K9 methylation, and gene expression.

## Materials and methods

### Strains, preservation and cultivation conditions

In this study, all strains were derived from *M. oryzae* 70–15, which was obtained from the American Type Culture Collection (ATCC, Manassas, VA, USA; accession number MYA-4616). For strain preservation, mycelium was stored on desiccated filter paper pieces at −20°C. For strain resuscitation, the filter paper pieces were placed on complete medium (CM) plates and incubated at 25°C. For vegetative growth and conidiation, small fungal blocks were transferred to fresh medium plates and incubated at 25°C under a precisely controlled light-dark cycle, consisting of 16 hours of illumination followed by 8 hours of darkness [36]. To harvest mycelium for subsequent RNA and protein extraction, fungal blocks of approximately 1 cm^2^ were homogenized with steel balls at 60 Hz for 1 minute with the homogenizer (Wuhan Servicebio Technology Co., Ltd, China), and then cultured in liquid CM at 25°C for 36 h with shaking at 150 revolutions per minute (rpm) [37]. All primers used in this study are listed in Supplementary Table S1.

### Sequence alignment and phylogenetic analysis

The protein sequences of MoKmt1 and MoCul4 were downloaded from the National Center for Biotechnology Information (NCBI) database (https://www.ncbi.nlm.nih.gov/). Homologs of MoCul4 were retrieved via BLASTP (https://blast.ncbi.nlm.nih.gov) using our target sequence as the query. Multiple sequence alignment was performed and visualized in the CLC Main Workbench. Phylogenetic analysis was conducted based on the aligned sequences. A Neighbor-Joining phylogenetic tree was then reconstructed using the Bootstrap method with 1000 bootstrap replicates under the Poisson model to assess the reliability of the tree topology. The final tree was visualized and annotated in MEGA12. The sequence alignment and phylogenetic analysis of MoCul4 are shown in Supplementary Figures S1 and S2.

### Gene knockout and complementation

The knockout vectors for *MoKMT1* (*MGG_06852*) and *MoCUL4* (*MGG_14763*) were constructed using the high-throughput gene knockout method [38]. Briefly, 1.2-kb upstream (Up) and downstream (Dn) flanking sequences of each target gene were amplified using primers containing 15-bp overlapping ends (Supplementary Table S1). The Up fragment, sulfonylurea resistance gene (*SUR*, 2.8 kb), and Dn fragment were directionally cloned into the *Xba*I/*Hin*dIII-linearized pKO1B vector through seamless recombination. Recombinant vectors were introduced into wild-type *M. oryzae* strain 70–15 through *Agrobacterium tumefaciens*-mediated transformation (ATMT). Transformants were selected on defined complex medium (yeast nitrogen base without amino acids 1.7 g/L; asparagine, 2 g/L; NH_4_NO_3_, 1 g/L; glucose, 10 g/L; pH adjusted to 6.0 with Na_2_HPO_4_) supplemented with 0.5 μM 5-fluoro-2’-deoxyuridine and 100 μg/mL sulfonylurea at 28°C. Putative mutants were confirmed by fluorescence identification and double PCR (Supplementary Figure S3 A-B), and the copy number of SUR was checked through qPCR (Supplementary Table S2) as described previously [6,38].

For complementation assays, a native genomic fragment containing the 1.5-kb native promoter region and 0.5-kb terminator region of *MoKMT1*/*MoCUL4* was amplified from wild-type genomic DNA. This fragment was ligated into the *Xba*I/*Hin*dIII-digested pKO1B-HPH vector. The complementation plasmid was transformed into the corresponding knockout mutants via ATMT. Complemented strains were selected on complete medium (CM) containing 200 μg/mL hygromycin B and verified by RT-PCR using gene-specific primers (Supplementary Table S1) [6,38].

### Growth and sporulation assays

For colony diameter detection, 4 mm mycelial blocks from the colony edge were inoculated onto 7 cm CM agar plates and cultured at 25°C for 7 days. The diameter was measured on the 8^th^ day with 5 biological replicates. For stress media assessment, 4 mm mycelial blocks from the colony edge were inoculated onto 7 cm CM agar plates supplemented with 0.005% SDS, 75 μg/mL CFW, 800 μg/mL Congo red, 8 mM H_2_O_2_, 1 mM methylviologen, 1 M D-sorbitol, 0.5 M NaCl, and 10 ng/mL Rapamycin and cultured at 25°C for 8 days. For sporulation, spores were collected with 3 mL sterile water, filtered through 3 layers of lens‑cleaning paper and counted via a hemocytometer with 5 biological replicates. The sporulation per unit area was calculated using the formula: Sporulation (spores/cm^2^) = (spore concentration × 3 mL) / colony area. Representative results are shown in the corresponding figures. For Western blot analysis, the experiments were repeated three times independently.

### Appressorium development and pathogenicity assays

For spore germination and appressorium formation assays, 40 μL of spore suspension (1 × 10^5^ spores/mL) was dropped onto PVC membranes at 25°C under humid conditions. Germination rate was assessed at 4 h, and appressorium formation rate was quantified at 24 h by the microscope (Olympus CX31, Japan). More than 200 conidia were counted with three biological replicates per experiment [3]. For rice leaf spray pathogenicity, 3 mL of a suspension of 5 × 10^4^ conidia/mL in 0.2% gelatin was sprayed on rice seedlings of cultivar CO-39 [39]. After inoculation, the rice seedlings were first incubated in the dark at 22°C under high humidity for 48 h and then transferred to an incubator at 25°C with a 16-h light/8-h dark photoperiod for an additional 4–5 days. Then at least fourteen diseased leaves were imaged for each strain and the lesion area was measured using Photoshop as described [40]. Briefly, a 5 cm segment was excised from each diseased leaf, and the total leaf area and healthy (green) leaf area were measured using the “Magic Wand” tool (tolerance set to 35) in Photoshop. The lesion area was calculated as total leaf area minus healthy leaf area, and the percentage of lesion area was calculated as (lesion area / total leaf area) × 100%. For pathogenicity assays on leaves of barley (Hordeum vulgare), 4-mm mycelial blocks from the strains were inoculated on leaves and incubated in a humid box at 25°C for 4 days [6]. At least two barley leaves were inoculated and imaged for each strain. The lesion area on barley leaves (approximately 1 cm in length) was calculated using the same method as described for rice leaves. Representative results are shown in the corresponding figures.

### Subcellular localization

To construct vectors expressing GFP fused to the N-terminus of MoCul4 and MoKmt1, the coding sequences of *MoCUL4* and *MoKMT1* were amplified and cloned into *Xba*I-linearized pKD8-GFP (which carried the G418 resistance gene *NEO* as a selectable marker) by using ClonExpress II One-Step Cloning Kit (Vazyme, Nanjing, China). The expression of GFP-MoCul4 and GFP-MoKmt1 fusion proteins was driven by the *SOD1* promoter and terminated by the *RP27* terminator. The recombinant plasmids were transformed into wild-type strain via ATMT. The transformants were screened on CM plates supplemented with 800 μg/mL G418, 400 μg/mL cefotaxime, and 60 μg/mL streptomycin. Subsequently, the pKD3-MoH2B-mCherry vector, which expressed mCherry fused to the C-terminus of histone H2B under the control of *H3* promoter and *RP27* terminator, was transformed into transformants expressing GFP-MoCul4 or GFP-MoKmt1. The transformants were screened on CM plates supplemented with 800 μg/mL bialaphos, 400 μg/mL cefotaxime, and 60 μg/mL streptomycin. Then the subcellular localizations of GFP-MoCul4/GFP-MoKmt1 and MoH2B-mCherry in conidia, appressoria and hyphae were observed through a confocal microscope (Nikon AI+ MMI CellManipulator Plus, Japan).

### Analysis of MoKmt1 expression

To detect the expression level of MoKmt1 in the wild type and Δ*Mocul4*, an approximately 1.5 kb native promoter region was amplified from the *M. oryzae* genome, and the GFP-MoKmt1 fusion fragment along with the *RP27* terminator was amplified from pKD8-GFP-MoKmt1. These two fragments were then ligated into *Xba*I/*Hin*dIII-linearized pKO1B-HPH. The resulting recombinant vector was introduced into the wild type strain 70–15 and the Δ*Mocul4* mutant, and transformants were selected on medium containing 200 μg/mL hygromycin B. The expression level of GFP-MoKmt1 was detected by Western blot.

### Western blot assay

For the detection of H3K9me3 level in mutants and GFP-MoKmt1 level in Δ*MoCul4*, the total proteins were extracted by homogenizing 0.2 g mycelium in 1 mL of protein extraction buffer (50 mM Tris-HCl, pH 7.5, 100 mM NaCl, 5 mM EDTA, 1% Triton X-100). Protein concentration was quantified using a Nano-300 micro-spectrophotometer (Hangzhou Ausheng Instrument Co., Ltd., Hangzhou, China). A total of 50 μg protein was separated on 10% SDS-PAGE gels (#PG112, Epizyme, Shanghai, China) and transferred onto polyvinylidene difluoride (PVDF) membranes for Western blot analysis. Then the PVDF membrane was blocked with 5% nonfat milk in TBST buffer (20 mM Tris-HCl, pH 7.5, 150 mM NaCl, 0.1% Tween-20) for 1 h at room temperature. The H3K9me3 level was assessed by anti-Histone H3 (trimethyl K9) antibody (#ab176916, Abcam, UK). The GFP-MoKmt1 level was detected by Anti-GFP antibody (# ET1607-31, Huabio, Hangzhou, China). The GAPDH antibody (#HA721131, Huabio, Hangzhou, China) and H3 antibody (#AF0863, Affinity Biosciences, USA) were used as loading controls. Then the secondary antibodies goat anti-rabbit IgG HRP (#S0001, Affinity Biosciences, USA) and goat anti-mouse IgG HRP (#S0002, Affinity Biosciences, USA) were used to probe the blot. All primary and secondary antibodies were diluted 1:1000 in TBST containing 5% nonfat milk. Chemiluminescent signals were detected using a Tanon 4800 Multi chemiluminescence imaging system (Tanon Science & Technology, Shanghai, China). The enhanced chemiluminescence (ECL) substrate used was the Affinity™ ECL kit (Affinity Biosciences, USA).

### RNA-seq

Liquid-cultured mycelia were harvested and pulverized in liquid nitrogen. RNA was extracted using homogenizing 0.1 g of powder in 1 mL of TRIzol reagent (Takara, Japan), with three biological replicates per strain. The subsequent total RNA extraction, RNA quality verification, cDNA library, paired-end sequencing, sequence alignment and differential expression analysis were conducted by a commercial service provider (Shanghai Personal Biotechnology Co., Ltd., China). Briefly, RNA integrity was evaluated either using standard RNA-denaturing agarose gel electrophoresis or an Agilent 2100 Bioanalyzer with the RNA 6000 Nano Kit (Agilent Technologies Inc., Santa Clara, CA, USA). Strand-specific sequencing libraries were constructed from ≥1 μg of total RNA using the NEBNext Ultra II Directional RNA Library Prep Kit for Illumina. Briefly, poly(A) mRNA was enriched using oligo(dT) beads, fragmented, and reverse-transcribed into cDNA. The cDNA underwent end-repair, adenylation, and adapter ligation. Fragments of 400–500 bp were size-selected using AMPure XP beads, followed by PCR amplification and purification. Library quality and size distribution were assessed using an Agilent 2100 Bioanalyzer, and the effective concentration was quantified by quantitative PCR (qPCR). The cDNA libraries were normalized, pooled in equimolar amounts, and subjected to paired-end sequencing (PE150) on an Illumina novaseq xplus sequencing platform. Raw sequencing reads in FASTQ format were first processed using Fastp to remove adapter sequences, low-quality bases, and reads with an average base quality score below Q20. The resulting high-quality clean reads were then aligned to the *M. oryzae* reference genome (https://www.ncbi.nlm.nih.gov/datasets/genome/GCF_000002495.2/) using HISAT2 (http://ccb.jhu.edu/software/hisat2/index.shtml) with default parameters. Gene expression was quantified as raw read counts using HTSeq (v0.9.1). Differential expression analysis between comparative groups was performed using DESeq2 (v1.38.3). Genes with an absolute log2 fold change > 1 and a nominal *p*-value < 0.05 were defined as significantly differentially expressed. Enrichment analysis for GO and KEGG was conducted using cluster Profiler (v4.6.0) with a hypergeometric distribution (*p*-value < 0.05) to determine the principal biological functions of the differentially expressed genes.

### RNA isolation and quantitative real-time PCR

For RNA isolation of liquid-cultured mycelia, homogenate of 1 cm^2^ fungal blocks was cultured in liquid CM at 25°C for 36 h with shaking at 150 rpm. For RNA isolation of appressoria, about 7 × 10^7^ spores per replicate were collected and cultured for 4 h on hydrophobic plastic membranes. For RNA isolation of invasive hyphae, barley leaves were spot-inoculated with ten 20-μL droplets of spore suspension (5 × 10^5^ spores/mL) per replicate and incubated for 2 days. All experiments were conducted with three independent biological replicates. The total RNA was isolated by using the FastPure Universal Plant Total RNA Isolation Kit (RC411-01, Vazyme Biotech Co., Ltd., China), and cDNA was synthesized from 1 µg of total RNA using HyperScript™ IV RT SuperMix for qPCR (with gDNA wiper) (K1590, APExBIO Technology, USA). The qPCR was conducted by using HotStart™ Universal 2X Green qPCR Master Mix (K1170, APExBIO Technology, USA).

### Statistical analysis

Statistical differences between mutants and the wild type were analyzed using two-tailed Student’s t-test in GraphPad Prism 10 (v.10.1.2). Data are presented as the mean ± standard deviation (SD). Significance levels were set as *p < 0.05. Schematic diagram was generated using an online diagram editor (https://app.diagrams.net/).

## Results

### Identification, multiple sequence alignment, and domain analysis of MoCul4 and MoKmt1

**Figure 1.**
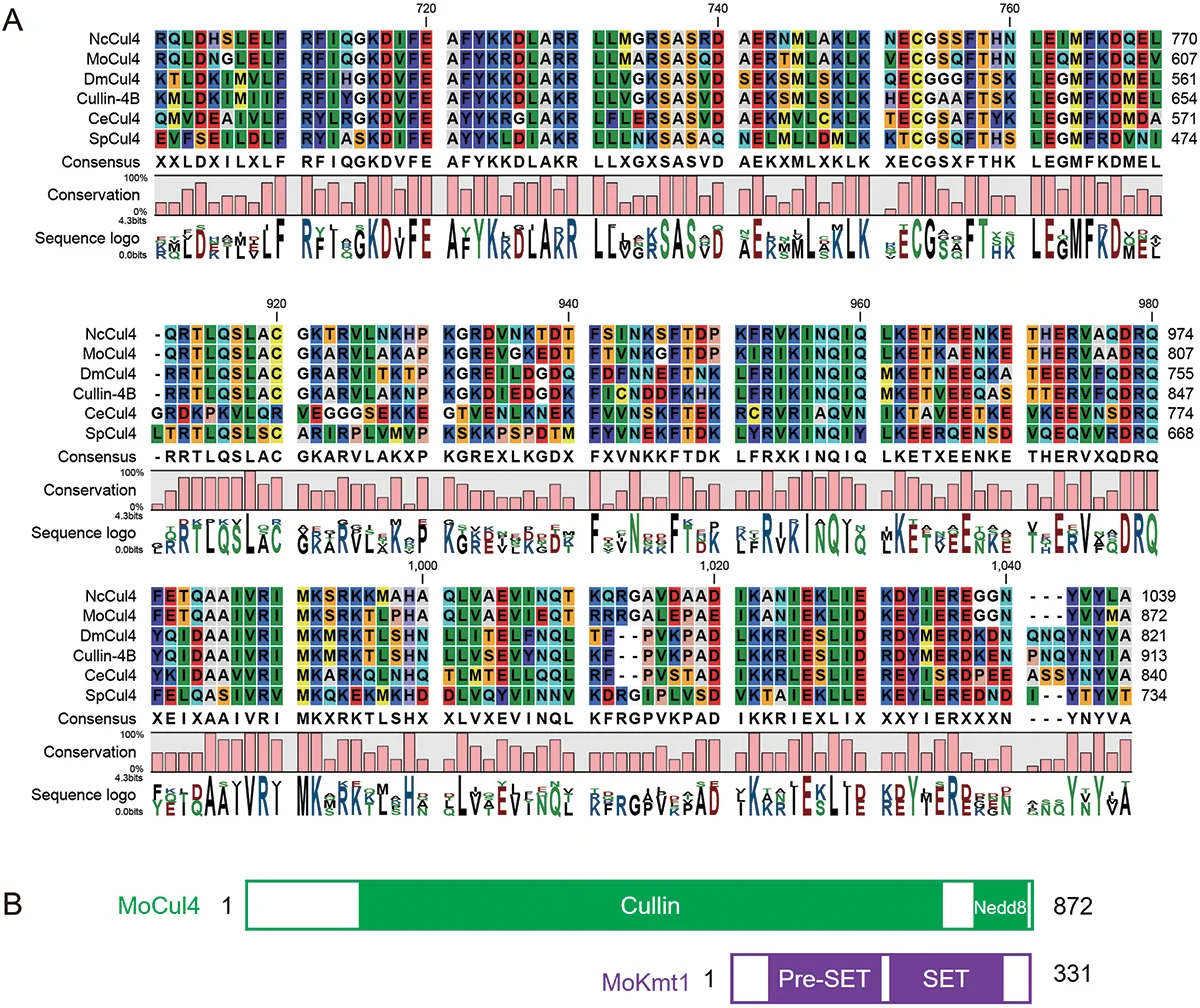
Multiple sequence alignment of MoCul4 and schematic diagrams of conserved domains in MoCul4 and MoKmt1. (A) Partial multiple sequence alignment of *Magnaporthe oryzae* MoCul4 with MoCul4 homologs from *Homo sapiens* (Cul4B), *Schizosaccharomyces pombe* (SpCul4), *Neurospora crassa* (NcCul4), *Caenorhabditis elegans* (CeCul4), and *Drosophila melanogaster* (DmCul4). The alignment corresponds to amino acid residues 540–607 in the Cullin domain and residues 739–872 in the Nedd8 domain of MoCul4. (B) Schematic diagram of conserved domains in MoCul4 and MoKmt1 of *M. oryzae*.

Homologs of *S. pombe* Cul4 and Clr4 were identified as MoCul4 (MGG_14763) and MoKmt1 (MGG_06852) in *M. oryzae*. The phylogenetic relationships of MoCul4 revealed that MoCul4 was clustered together with other fungi (such as *Aspergillus fumigatus*, *N. crassa*, *Fusarium graminearum*, and *S. pombe*), separated from the plant and animal clades, confirming their early evolutionary divergence of fungal Cul4 (Supplementary Figure S1). Yet multiple sequence alignment with MoCul4 homologs from *H. sapiens* (Cul4B), *S. pombe* (SpCul4), *N. crassa* (NcCul4), *C. elegans* (CeCul4), and *D. melanogaster* (DmCul4) revealed several conserved motifs (such as residues 540–607 and 739–872) among all analyzed species (Figure 1(A), see Supplementary Figure S2 for the complete multiple sequence alignment) which are located within the Cullin (126–772) and Nedd8 (807–867) domains of MoCul4 Figure 1(B), despite the presence of substitutions and gaps. MoKmt1 contains the pre-SET (40–166) and SET (175–302) domains Figure 1(B). The SET domain is known to exhibit catalytic activity in histone methylation. Based on the protein structural conservation and functional studies in other species, we suggest that MoCul4 and MoKmt1 are involved in H3K9 methylation in *M. oryzae*.

### *MoCul4 and MoKmt1 regulate vegetative growth and conidiation in* M. oryzae

**Figure 2.**
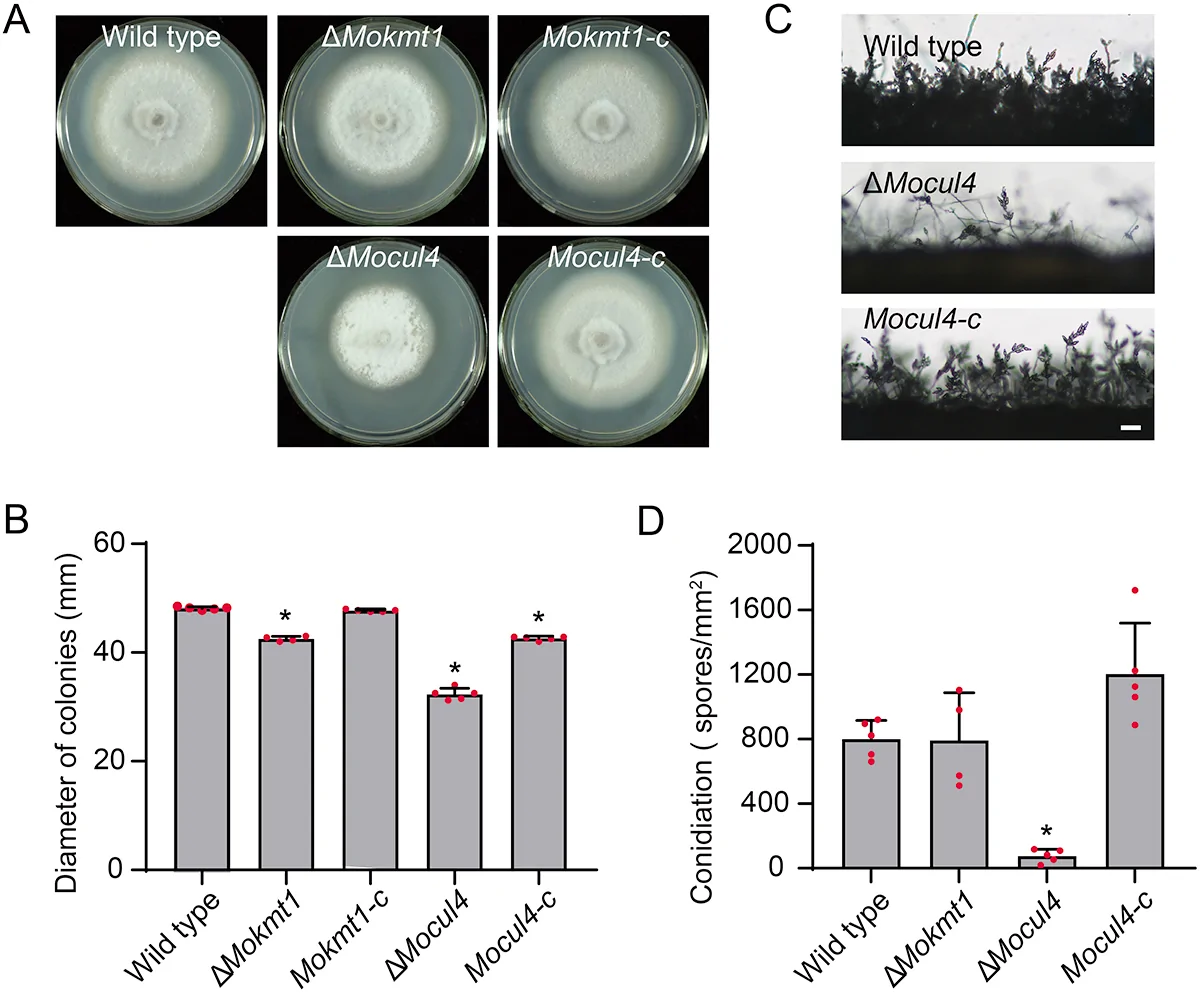
Growth and conidiation analysis of the mutants. (A, B) Colony morphology and diameter of mutants Δ*Mokmt1*, Δ*Mocul4*, and complemented strains *Mokmt1-c, Mocul4-c* (*n* = 4–5 biological replicates per strain). (C) Conidiophore morphology of mutant Δ*Mocul4* and complemented strain *Mocul4-c*. Scale bar = 50 μm. (D) Conidiation quantification of mutants Δ*Mokmt1*, Δ*Mocul4*, and complemented strain *Mokmt1-c* (*n* = 4–5 biological replicates per strain). All the experiments were independently repeated three times. Data are presented as means ± SD. Statistical significance was determined using two-tailed Student’s t-test (*p < 0.05 compared to the wild type).

The *MoKMT1* and *MoCUL4* genes were knocked out in *M. oryzae* via homologous recombination, and the null mutants (Δ*Mokmt1* and Δ*Mocul4*) were validated by double PCR (Supplementary Figure S3). Phenotypic analysis demonstrated that Δ*Mokmt1* (42.5 ± 0.5 mm) exhibited an 11.6% reduction in hyphal growth rate compared to the wild type (48.1 ± 0.3 mm), while Δ*Mocul4* (32.3 ± 1.1 mm) showed a more severe 32.8% decrease Figure 2(A,B). Although conidiation of Δ*Mokmt1* (792.8 ± 294.0 spores/mm^2^) remained unchanged, Δ*Mocul4* (76.5 ± 40.4 spores/mm^2^) displayed a 90.4% reduction relative to the wild type (801.0 ± 114.5 spores/mm^2^) Figure 2(D). The mutant Δ*Mocul4* exhibited significantly fewer conidiophores by microscopic observation, which directly explained its conidiation defect Figure 2(C). Genetic complementation strains (*Mokmt1-c* and *Mocul4-c*) fully restored both growth and conidiation Figure 2(A–D), confirming that both MoKmt1 and MoCul4 are involved in regulating growth and asexual reproduction in *M. oryzae*. Notably, the more severe defects in Δ*Mocul4* suggest MoCul4 has functions beyond its partnership with MoKmt1.

### *MoCul4 and MoKmt1 are involved in the pathogenicity of* M. oryzae

**Figure 3.**
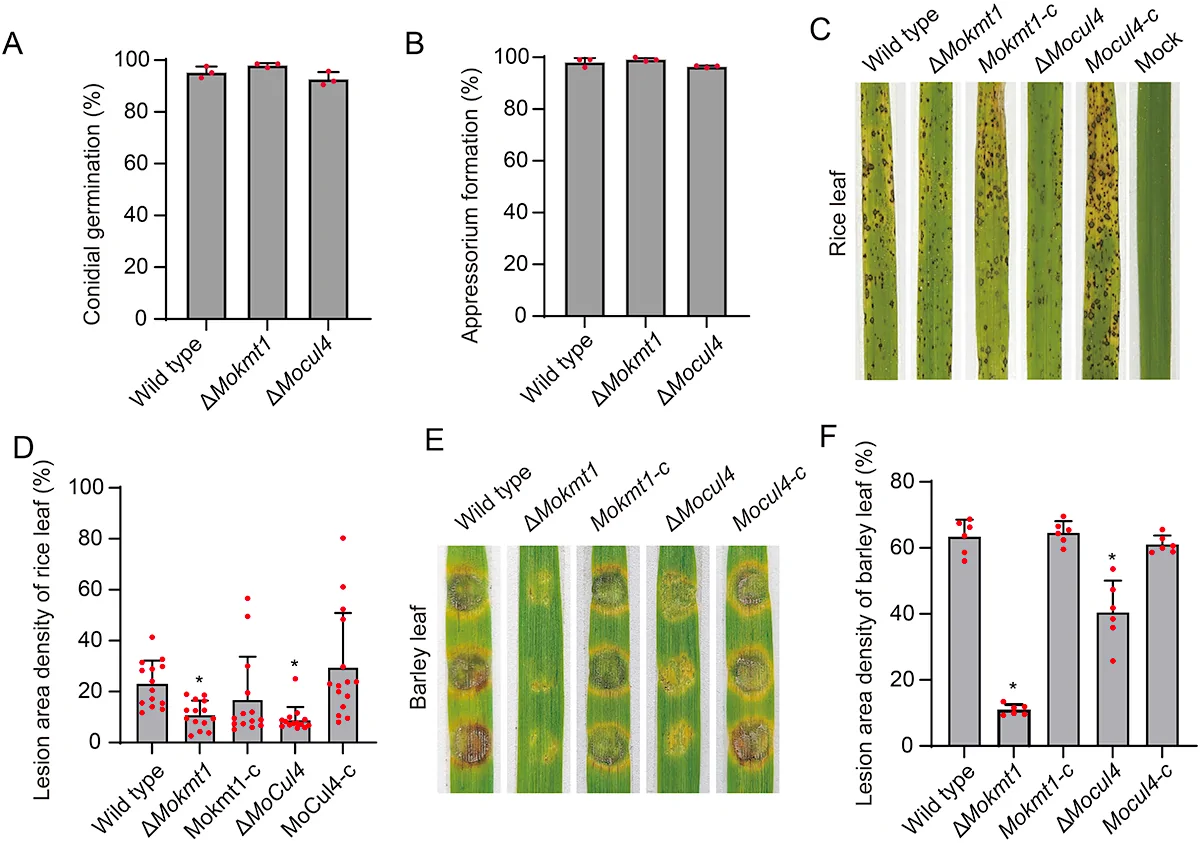
Analysis of conidial germination, appressorium formation, and pathogenicity in mutants. (A, B) Conidial germination rates and appressorium formation rates of mutants Δ*Mokmt1* and Δ*Mocul4* (*n* = 3 biological replicates per strain). (C) Pathogenicity analysis on rice leaves for mutants Δ*Mokmt1*, Δ*Mocul4*, and complemented strains *Mokmt1-c, Mocul4-c*. (D) The quantification of lesion areas on 5 cm rice leaf (*n* ≥ 14 biological replicates per strain). (E) Pathogenicity analysis on barley leaves for mutants Δ*Mokmt1*, Δ*Mocul4*, and complemented strains *Mokmt1-c, Mocul4-c*. (F) The quantification of lesion areas on 1 cm barley leaf (*n* = 6 biological replicates per strain). All the experiments were independently repeated three times. Data are presented as means ± SD. Statistical significance was determined using two-tailed Student’s t-test (*p < 0.05 compared to the wild type).

Analysis of conidial germination and appressorium formation rates revealed that these processes were unaffected by the deletion of *MoCUL4* or *MoKMT1* Figure 3(A,B). Rice leaf spray inoculation assays demonstrated that the virulence of both Δ*Mokmt1* (11.0 ± 5.4%) and Δ*Mocul4* (9.1 ± 4.9%) was significantly reduced, with lesion areas decreased by more than 50% compared with the wild type (23.1 ± 9.1%) Figure 3(C,D). Consistent with rice leaf pathogenicity assays, the barley leaf pathogenicity assays also showed that the mutants Δ*Mokmt1* and Δ*Mocul4* exhibited reduced virulence, which was largely rescued by genetic complementation Figure 3(E,F). These results indicate that while MoCul4 and MoKmt1 are dispensable for conidial germination and appressorium development, they are essential for full pathogenicity of *M. oryzae*, mainly because the invasive growth of the mutants was defective.

### *MoCul4 and MoKmt1 localize in the nucleus and are required for H3K9 methylation in* M. oryzae

**Figure 4.**
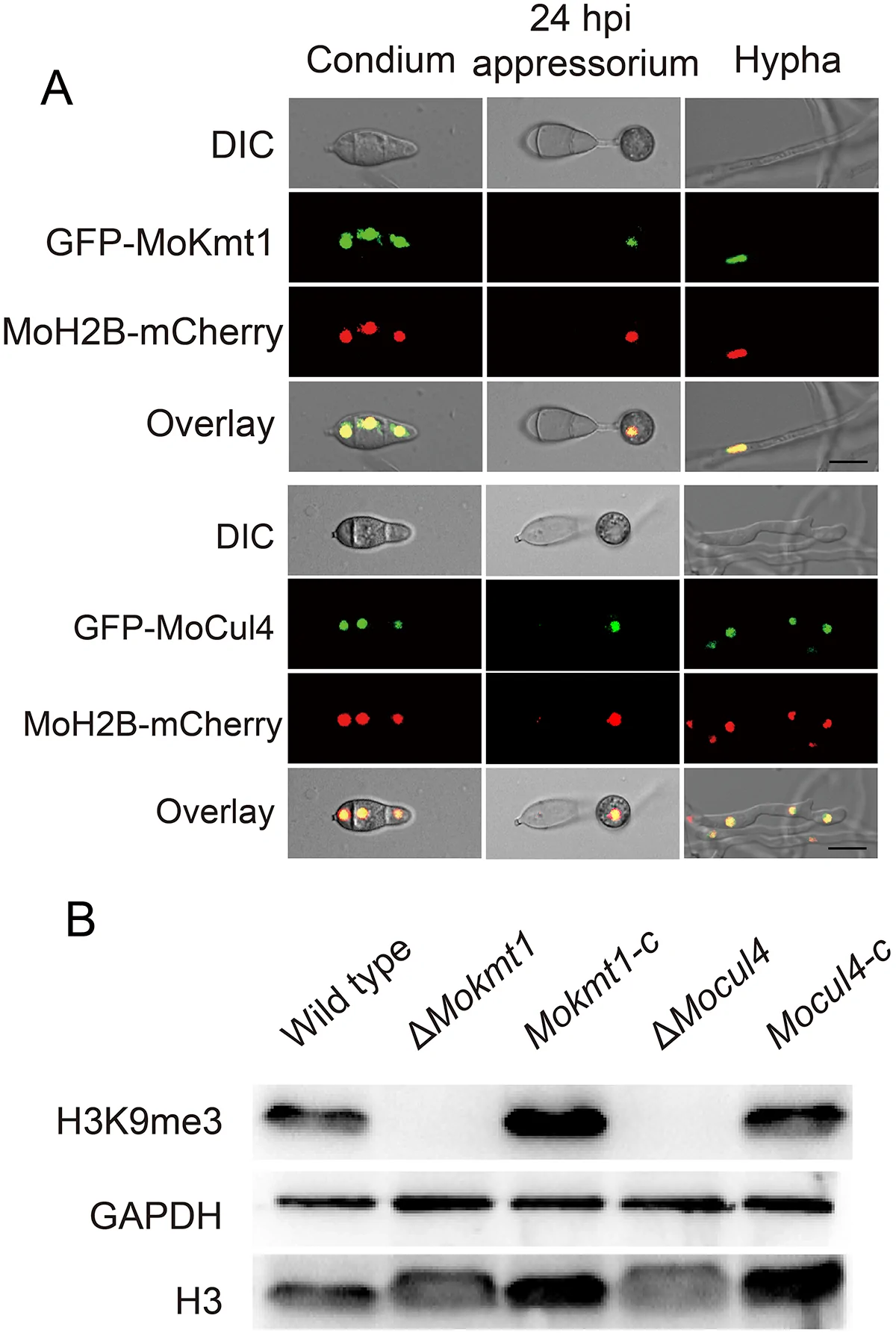
The subcellular localization of MoCul4 and MoKmt1 and analysis of H3K9me3 levels in mutants. (A) The subcellular co-localization of GFP-MoCul4/MoKmt1 and MoH2B-mCherry in conidia, appressoria, and hyphae. Scale bar = 10 μm. (B) Western blot analysis of H3K9me3 levels in Δ*Mokmt1*, Δ*Mocul4*, and complemented strains *Mokmt1-c* and *Mocul4-c*.

To determine the subcellular distribution, we observed the localization of GFP-MoCul4/MoKmt1 and the nuclear marker MoH2B-mCherry, and found that both MoCul4 and MoKmt1 were predominantly localized in the nucleus Figure 4(A). To investigate whether MoCul4 and MoKmt1 regulate H3K9 methylation, we analyzed H3K9me3 levels in mutant mycelia via Western blotting. The Δ*Mocul4* and Δ*Mokmt1* mutants exhibited near-complete loss of H3K9me3 Figure 4(B). Complementation of *MoCUL4* and *MoKMT1* restored H3K9me3 to wild-type levels in both strains. To assess whether MoCul4 affects MoKmt1 expression and degradation, we measured *MoKMT1* expression by qPCR and found no significant difference between the mutant and wild‑type strains (Supplementary Figure S4A). Furthermore, a native promoter‑driven GFP‑MoKmt1 construct was transformed into wild‑type and Δ*Mocul4* backgrounds, and no obvious difference in GFP‑MoKmt1 expression was observed (Supplementary Figure S4B). Deletion of *MoCUL4* does not alter the mRNA or protein levels of MoKmt1, indicating that MoCul4 does not regulate MoKmt1 expression or stability. These results demonstrate that MoCul4 localizes in the nucleus and is essential for MoKmt1-mediated H3K9 methylation in *M. oryzae*.

### MoCul4 and MoKmt1 mediate heterochromatic gene silencing and global transcriptional regulation

**Figure 5.**
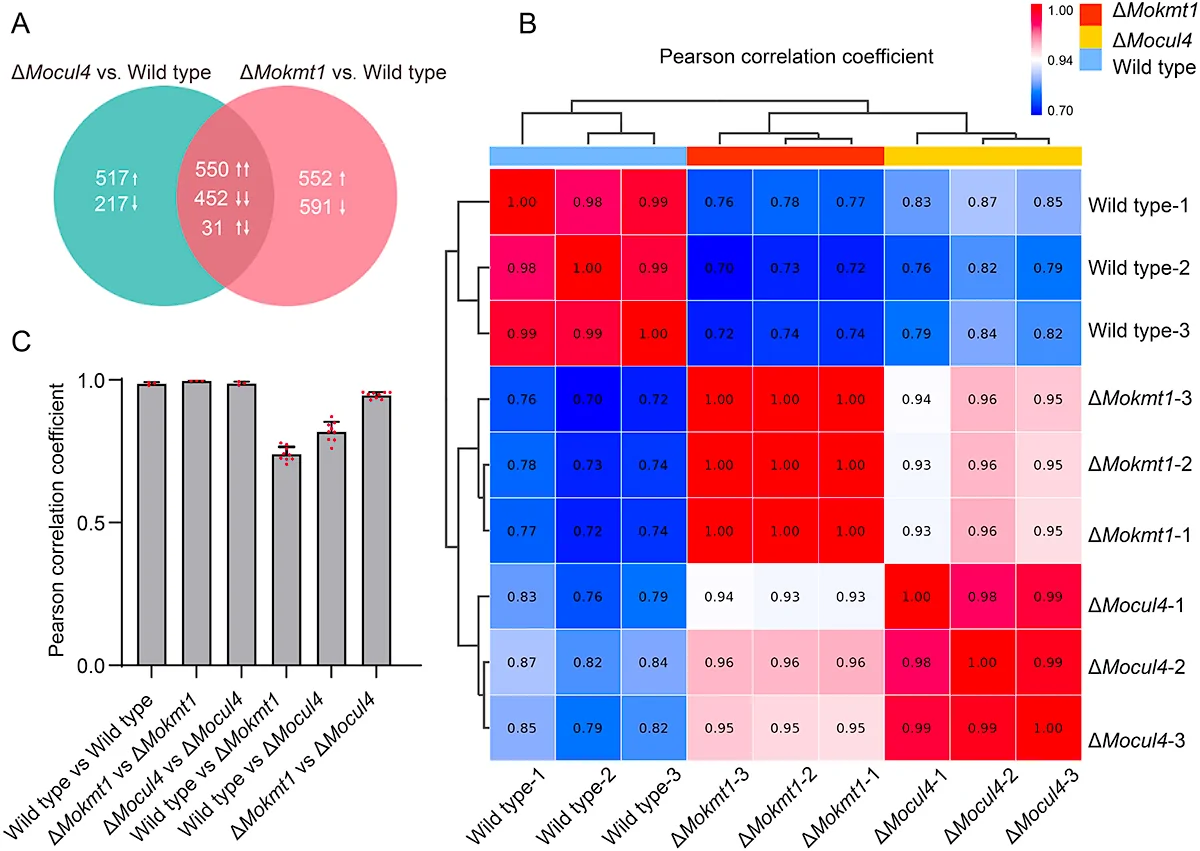
Analysis of co‑regulated genes and sample correlations. (A) Venn diagram of differentially expressed genes identified in Δ*Mocul4* and Δ*Mokmt1* compared to the wild type based on RNA-seq data. (B) Pearson correlation heatmap of three biological replicates per strain (wild type, Δ*Mokmt1*, Δ*Mocul4*). The color gradient from blue (low correlation) to red (high correlation) allows visual comparison. (C) Statistical summary of the Pearson correlation coefficients. Bar graphs with overlaid individual data points show the distribution of *r* values for intra‑group comparisons and inter‑group comparisons.

H3K9me3 is a hallmark modification of heterochromatin (gene repression) [21]. In the mutants Δ*Mocul4* and Δ*Mokmt1*, the H3K9me3 levels were almost completely abolished. To further verify whether MoKmt1 and MoCul4 were involved in the formation of heterochromatin and consequently regulated the gene expression patterns of *M. oryzae*, we analyzed gene expression profiles in the mycelia of Δ*Mocul4* and Δ*Mokmt1* mutants via RNA-seq. Our results indicated a high overlap in differentially expressed genes (DEGs) between the two mutants Figure 5(A) with 1,033 common DEGs, accounting for 58.5% of the total DEGs (1,767) in the Δ*Mokmt1* mutant, and 47.5% of the total DEGs (2,176) in the Δ*Mocul4* mutant. Among the common DEGs, 550 DEGs were co‑upregulated, 452 were co‑downregulated, and 31 showed opposite regulation patterns between the two mutants (the overlapping DEGs, as well as DEGs unique to the Δ*Mocul4* and Δ*Mokmt1* mutants, are listed in Supplementary Tables S3-S5, respectively).

To assess the correlation between the mutants, we examined the Pearson correlation coefficient matrix, which reflects the similarity of gene expression profiles among samples. A coefficient closer to 1 indicates higher similarity in expression patterns between samples. Generally, correlation coefficients between 0.8 and 1 are considered strongly correlated. Before performing differential expression analysis, checking the gene expression correlation between samples is an important step to assess experimental reliability and the validity of sample selection. The results showed that the average correlation coefficients among the three biological replicates of the wild-type, Δ*Mokmt1*, and Δ*Mocul4* strains were 0.98 ± 0.01, 1.00 ± 0.00, and 0.99 ± 0.01, respectively (*n* = 3), all > 0.98, indicating strong intragroup consistency. Compared with the wild-type group, samples from the Δ*Mokmt1* and Δ*Mocul4* groups showed relatively low inter-sample correlations (0.74 ± 0.03, *n* = 9; and 0.82 ± 0.03, *n* = 9, respectively), suggesting that gene knockout induces substantial expression divergence. The coefficient between the Δ*Mokmt1* and Δ*Mocul4* mutants was 0.95 ± 0.01 (*n* = 9), indicating high similarity Figure 5(B,C). Together, these results demonstrate that the gene expression profiles of all samples are highly reproducible, and that the two mutants show strong consistency in their overall expression patterns, suggesting the functions of MoKmt1 and MoCul4 are highly consistent.

**Figure 6.**
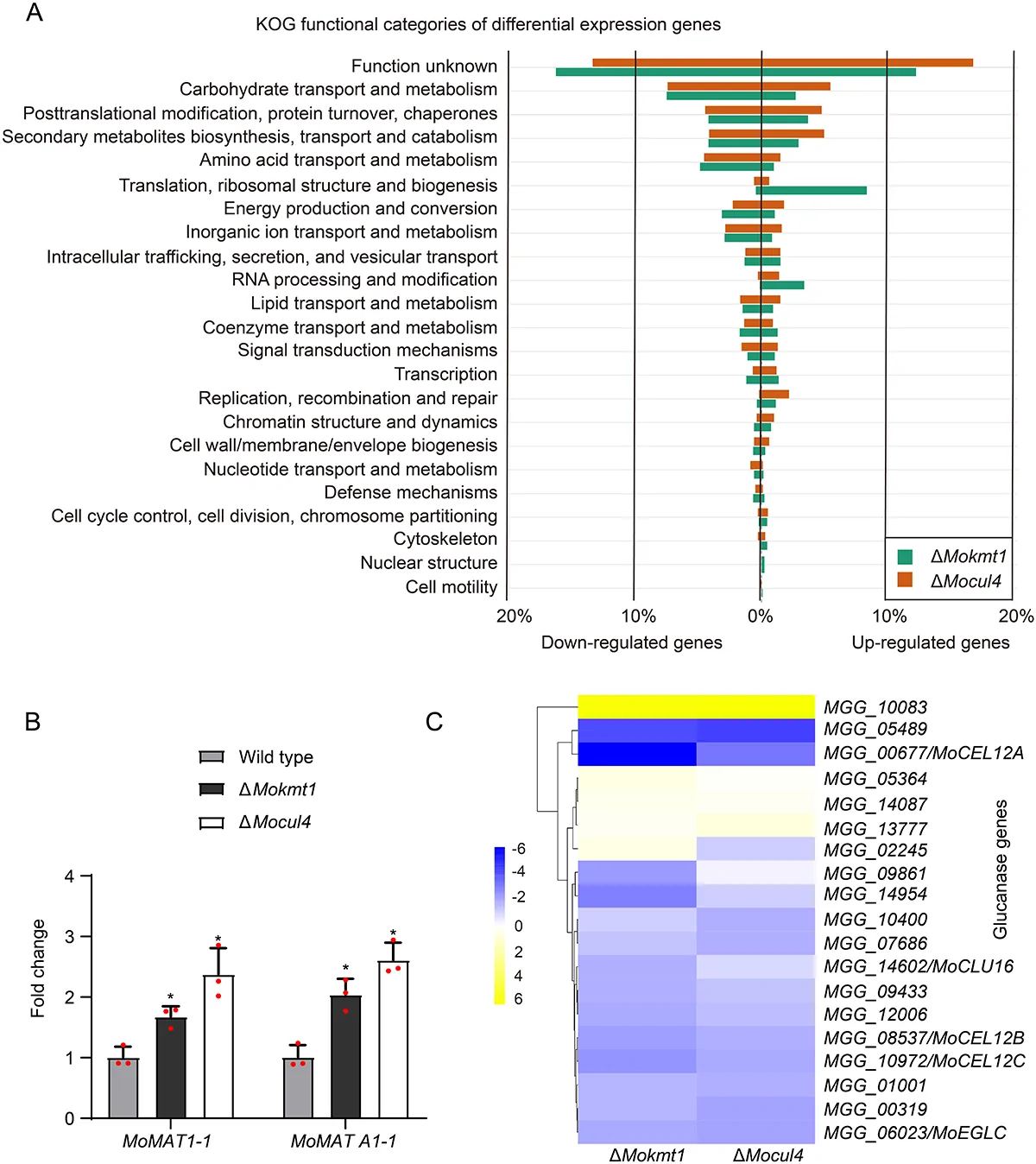
Functional annotation and expression level of genes co‑regulated by MoKmt1 and MoCul4. (A) KOG classification of the differentially expressed genes in Δ*Mocul4* and Δ*Mokmt1*. The bar chart shows the percentage of DEGs assigned to each functional category. (B) qPCR results for *MoMAT1-1* (*MGG_02979*) and *MoMAT A1-1 (MGG_02978*), located in heterochromatin regions, showing significant upregulation in the mutants compared to wild type (*n* = 3 biological replicates per strain). Gene expression levels were normalized to the *β-TUBULIN* and *H3* reference genes and were shown relative to the wild type. Data are presented as means ± SD. Statistical significance was determined using two-tailed Student’s t-test (*p < 0.05 compared to the wild type). (C) Heatmap analysis of β-glucanase genes showing ≥ 2-fold up- or down-regulation in Δ*Mocul4* and Δ*Mokmt1*.

Compared to Δ*Mokmt1*, the KOG functional classification of differentially expressed genes (DEGs) in Δ*Mocul4* exhibited high overall consistency (all the KOG functional classifications and GO enrichment of DEGs in Δ*Mocul4* and Δ*Mokmt1* mutants were listed in Supplementary Tables S6-8). However, in the two specific functional categories of “translation, ribosomal structure and biogenesis” and “RNA processing and modification,” a large number of genes were significantly up-regulated in Δ*Mokmt1* Figure 6(A). We further analyzed the expression levels of *MoMAT1-1* (*MGG_02979*) and *MoMAT A1-1* (*MGG_02978*), which are located in heterochromatin. The result showed that the expression level of *MoMAT1-1* significantly increased approximately twofold in the mutant Δ*Mokmt1*, while the expression level of *MoMAT A1-1* significantly increased more than two-fold in both mutants Figure 6(B). Furthermore, the heatmap analysis revealed that the expression of β-glucanase genes was significantly down-regulated in both Δ*Mokmt1* and Δ*Mocul4* Figure 6(C), which fundamentally participate in the cell wall remodeling and pathogenesis and plant immune suppression of *M. oryzae*. Additionally, we collected RNA at the 4 hpi appressorium and invasive hyphae stages and performed qPCR validation on several pathogenesis-related genes. The results showed that at the mycelial stage, generally consistent with the RNA-seq data, the expression patterns of several transcription factors and glucanase genes were all downregulated. At the appressorium and invasive hyphae stages, except for the *MoHOX2* gene, which exhibited differential expression trends (upregulated in the Δ*Mokmt1* mutant but downregulated in the Δ*Mocul4* mutant), all other detected pathogenesis-related genes displayed highly consistent expression trends in both mutants (Supplementary Figure S5). These findings collectively indicate that these genes are co-regulated by MoCul4 and MoKmt1. However, this regulation is likely indirect. The loss of MoKmt1 and MoCul4 function leads to defective heterochromatin assembly, resulting in global alterations in chromatin structure and indirectly disrupting the normal expression of these downstream genes. Such aberrant regulation of pathogenesis-related gene expression may be the underlying mechanism for the reduced pathogenicity observed in the mutants.

## Discussion

Histone PTMs regulate gene expression by altering chromatin structure, thereby modulating the access of transcriptional machinery to DNA [41]. Among these, histone H3 lysine 9 methylation (H3K9me3) is a hallmark of heterochromatin [21]. These regions are typically highly condensed, exhibit elevated H3K9 methylation levels, and exhibit low transcriptional activity. Conversely, histone H3 lysine 4 methylation (H3K4me) and histone H3 acetylation are characteristic marks of euchromatin and are predominantly localized to transcriptionally active regions [8].

In *M. oryzae*, transcriptional regulation research has historically centered on transcription factors, with systematic characterization of Zn_2_Cys_6_ zinc finger [38], C2H2 zinc finger [6], and homeobox transcription factors [42] demonstrating their indispensable roles in growth, sporulation, appressorium maturation, and pathogenesis. Recently, epigenetic regulation through histone modification-mediated chromatin remodeling has emerged as a major focus, revealing critical functions of H3K4 methylation (catalyzed by MoSet1 complex and reversed by MoJmj1) [19,20,43], H3K27 methylation (mediated by the PRC2 complex) [22,23], and H3 deacetylation (modulated by the Tig1 HDAC complex) [17]. Disruption of these crucial histone-modifying enzymes consistently causes severe defects in fungal development and virulence, underscoring their essential roles. In contrast, H3K9me3, a classic hallmark of constitutive heterochromatin, remains poorly characterized in *M. oryzae*. It not only maintains genomic stability and prevents aberrant recombination by silencing repetitive sequence regions such as centromeres and telomeres, but also recruits HP1 family proteins and other effectors to promote chromatin compaction, thereby establishing a stable transcriptional repressive environment that keeps target genes in a long‑term silenced state [44]. Collectively, these functions ensure the stability of higher‑order chromatin architecture and normal genome function. In *M. oryzae*, MoKmt1 has been identified as the sole methyltransferase whose deletion abolishes H3K9me3 entirely [19]. However, its regulatory mechanisms and functional partners remain unresolved.

The H3K9 methylation mechanism is highly conserved across species. In *H. sapiens*, *S. pombe*, and *N. crassa*, Cul4 and its homologues are required for H3K9 methylation catalyzed by DNMT3A/Clr4/Dim-5. Cul4 also serves as the scaffold of the Cullin‑RING Ligase 4 (CRL4) E3 ubiquitin ligase. In *M. oryzae*, the MoCul4 interacts with the ubiquitination suppressor MoCand2 and negatively regulates autophagy [35]. In this study, we further revealed the physiological functions of MoCul4 in establishing H3K9me. Phylogenetic analysis revealed that MoCul4 exhibited a shorter genetic distance to other filamentous fungi (such as *N. crassa* and *F. graminearum*) and *S. pombe* than to animals and plants (Supplementary Figure S1). Based on multiple sequence alignment, we found MoCul4 (*M. oryzae*) has a high identity with Cul4B (*H. sapiens*), SpCul4 (*S. pombe*), NcCul4 (*N. crassa*), CeCul4 (*C. elegans*), and DmCul4 (*D. melanogaster)* (Figure 1(A), Supplementary Figure S2). We therefore hypothesized that MoCul4 assists MoKmt1 in catalyzing H3K9 methylation. To verify this, we measured H3K9me3 levels in mutants and subcellular localization. As predicted, MoCul4 and MoKmt1 were both localized in nucleus and H3K9me3 levels were significantly decreased in mutants Δ*Mocul4* and Δ*Mokmt1* Figure 4(A,B), confirming that MoCul4 is essential for MoKmt1 to perform its H3K9 methyltransferase function. Consistent with prior reports in the Br48 strain [19], the Δ*Mokmt1* phenotype was identical in our experiments using the wild-type strain 70–15, including reduced hyphal growth, attenuated virulence, and decreased H3K9me3 levels, while conidiation, conidial germination, and appressorium formation remained unaffected. Phenotypic analyses showed parallel defects in both mutants, such as significant reduction in hyphal growth Figure 2(A,B), unimpaired conidial germination and appressorium formation Figure 3(A,B), but markedly diminished pathogenicity Figure 3(C,F). *Mocul4-c* showed a growth and conidiation restoration, yet the level was significantly different from that of wild type, which may be attributed to positional effects from random integration, which can influence exogenous gene expression. These results establish that MoCul4 and MoKmt1 co-regulate growth, development, and pathogenesis in *M. oryzae*. Not only do MoCul4 and MoKmt1 show strong consistency in phenotype, but they also exhibit high consistency in the regulatory patterns of gene expression. RNA-seq analysis showed that the differentially expressed genes (DEGs) shared by Δ*Mocul4* and Δ*Mokmt1* accounted for 58.5% of total DEGs in Δ*Mocul4* and 47.5% in Δ*Mokmt1*, respectively Figure 5(A). The Pearson correlation coefficient between Δ*Mocul4* and Δ*Mokmt1* was also high Figure 5(B). Moreover, the expression levels of glucanase genes and transcriptional factors in Δ*Mocul4* and Δ*Mokmt1* were largely consistent across all developmental stages examined (hyphae, appressoria and invasive hyphae) (Supplementary Figure S5), supporting a functional association between MoCul4 and MoKmt1. Given the established link between H3K9me3 and heterochromatin, we examined the expression of heterochromatin-associated genes *MoMAT1-1* and *MoMAT A1-1*. Both genes were upregulated in Δ*Mocul4* and Δ*Mokmt1* mutants Figure 6(B), indicating compromised heterochromatin integrity. However, the precise mechanism by which MoCul4 assists MoKmt1 remains elusive and requires further investigation.

**Figure 7.**
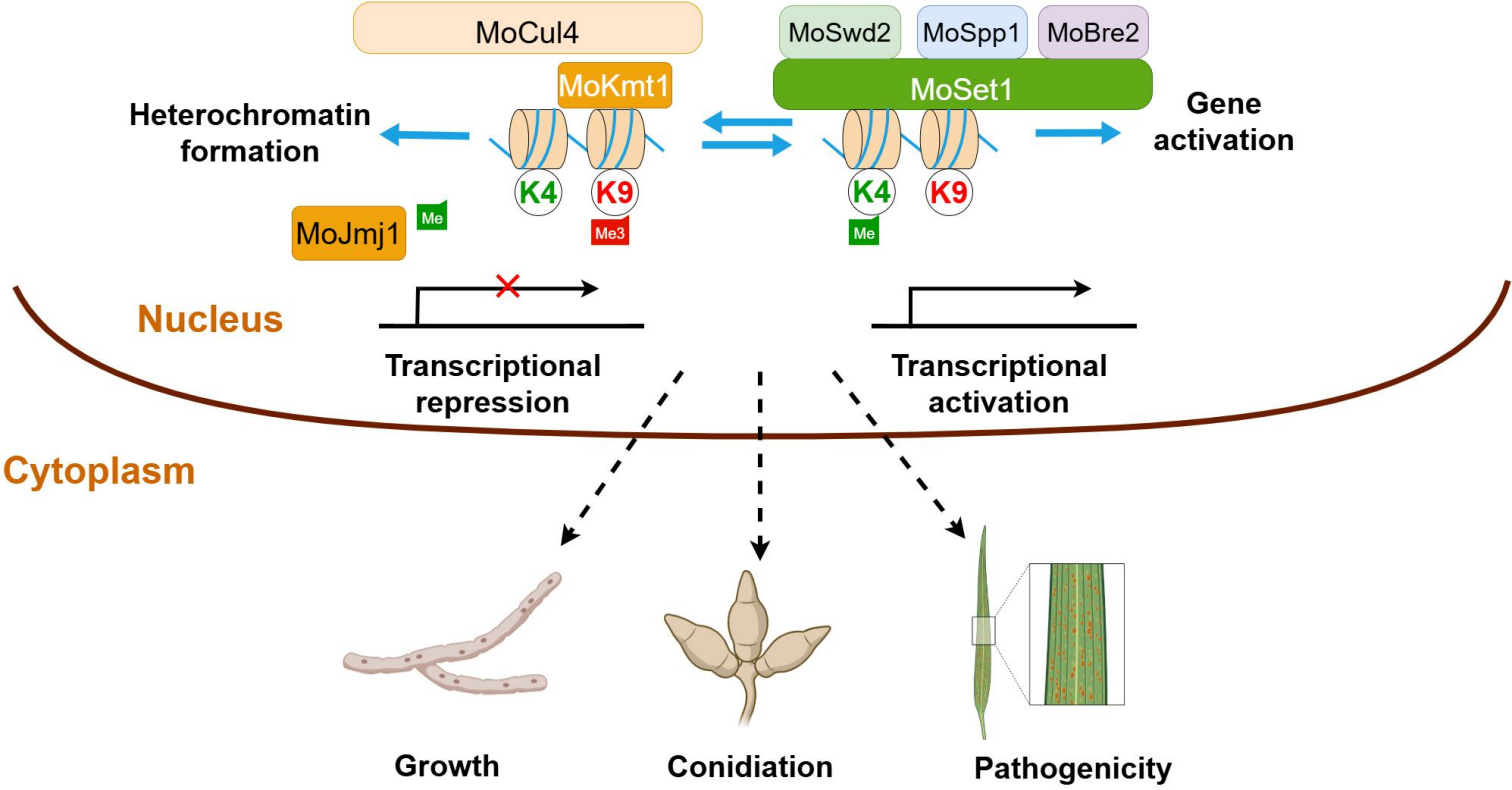
Schematic diagram of MoCul4 and MoKmt1 functions in *Magnaporthe oryzae*.

This study demonstrates that MoCul4, coordinated with MoKmt1, serves as an essential factor for MoKmt1 to catalyze H3K9 methylation and thereby promotes heterochromatin formation. Thus, MoCul4 and MoKmt1 modulate gene expression and participate in growth, development, and pathogenesis of *M. oryzae* (Figure 7). However, whether additional cofactors contribute to MoKmt1-mediated functions, as well as the genes directly targeted by MoKmt1, remain to be elucidated. Our research on this model phytopathogen not only provides a theoretical foundation for developing epigenetic-targeting agents to control *M. oryzae*, but also offers a valuable reference for studying other fungal pathogens.

## Supplementary Material

Supplemental Material

Supplementary Figure S1.jpg

Supplementary Figure S2 1.jpg

Supplementary Figure S2 3.jpg

Supplementary Figure S2 2.jpg

Supplementary Figure S5.jpg

Supplementary Figure S4.jpg

Supplementary Figure S3.jpg

Discription of figure.docx

## Data Availability

The raw sequencing data are available at the Genome Sequence Archive (GSA) under accession numbers CRA027197 (https://ngdc.cncb.ac.cn/gsa/search?searchTerm=CRA027197, MoClr4 refers to MoKmt1) [45]. The analyzed data supporting the findings (DEGs, GO enrichment, etc.) are available at the National Genomics Data Center (NGDC) under accession numbers OMIX012048 (https://ngdc.cncb.ac.cn/omix/release/OMIX012048, MoClr4 refers to MoKmt1) [46]. The raw data supporting the findings of this study are openly available in Figshare at https://doi.org/10.6084/m9.figshare.31440472 [47].
