# Supplementary material for: MoCul4 assists MoKmt1 to catalyze H3K9 methylation and regulates the growth, development, and pathogenicity in Magnaporthe oryzae
Source: Virulence. 2026 Sep 11;17(1):2715212. doi: 10.1080/21505594.2026.2715212 (PMC13577262; doi:10.1080/21505594.2026.2715212)
Supplement: Supplementary file 1 [file KVIR_A_2715212_SM1962.docx]

**Figure 1. Multiple sequence alignment of MoCul4 and schematic diagrams of conserved domains in MoCul4 and MoKmt1.** (A) Partial multiple sequence alignment of *Magnaporthe oryzae* MoCul4 with MoCul4 homologs from *Homo sapiens* (Cul4B), *Schizosaccharomyces pombe* (SpCul4), *Neurospora crassa* (NcCul4), *Caenorhabditis elegans* (CeCul4), and *Drosophila melanogaster* (DmCul4). The alignment corresponds to amino acid residues 540–607 in the Cullin domain and residues 739–872 in the Nedd8 domain of MoCul4. (B) Schematic diagram of conserved domains in MoCul4 and MoKmt1 of *M. oryzae*.

**Figure 2. Growth and conidiation analysis of the mutants.** (A, B) Colony morphology and diameter of mutants Δ*Mokmt1*, Δ*Mocul4*, and complemented strains *Mokmt1-c*, *Mocul4-c* (n = 4–5 biological replicates per strain). (C) Conidiophore morphology of mutant Δ*Mocul4* and complemented strain *Mocul4-c*. Scale bar = 50 μm. (D) Conidiation quantification of mutants Δ*Mokmt1*, Δ*Mocul4*, and complemented strain *Mokmt1-c* (n = 4–5 biological replicates per strain). All the experiments were independently repeated three times. Data are presented as means ± SD. Statistical significance was determined using two-tailed Student’s t-test (**P* < 0.05 compared to the wild type).

**Figure 3. Analysis of conidial germination, appressorium formation, and pathogenicity in mutants.** (A, B) Conidial germination rates and appressorium formation rates of mutants ΔMokmt1 and ΔMocul4 (n = 3 biological replicates per strain). (C) Pathogenicity analysis on rice leaves for mutants ΔMokmt1, ΔMocul4, and complemented strains *Mokmt1-c*, *Mocul4-c.* (D) The quantification of lesion areas on 5 cm rice leaf (n ≥ 14 biological replicates per strain)*.* (E) Pathogenicity analysis on barley leaves for mutants ΔMokmt1, ΔMocul4, and complemented strains *Mokmt1-c*, *Mocul4-c.* (F) The quantification of lesion areas on 1 cm barley leaf (n = 6 biological replicates per strain). All the experiments were independently repeated three times. Data are presented as means ± SD. Statistical significance was determined using two-tailed Student’s t-test (**P* < 0.05 compared to the wild type).

**Figure 4. The subcellular localization of MoCul4 and MoKmt1 and analysis of H3K9me3 levels in mutants.** (A) The subcellular co-localization of GFP-MoCul4/MoKmt1 and MoH2B-mCherry in conidia, appressoria, and hyphae. Scale bar = 10 μm. (B) Western blot analysis of H3K9me3 levels in Δ*Mokmt1*, Δ*Mocul4*, and complemented strains *Mokmt1-c* and *Mocul4-c*.

**Figure 5. Analysis of co‑regulated genes and sample correlations.** (A) Venn diagram of differentially expressed genes identified in Δ*Mocul4* and Δ*Mokmt1* compared to the wild type based on RNA-seq data. (B) Pearson correlation heatmap of three biological replicates per strain (wild type, Δ*Mokmt1*, Δ*Mocul4*). The color gradient from blue (low correlation) to red (high correlation) allows visual comparison. (C) Statistical summary of the Pearson correlation coefficients. Bar graphs with overlaid individual data points show the distribution of r values for intra‑group comparisons and inter‑group comparisons.

**Figure 6. Functional annotation and expression level of genes co‑regulated by MoKmt1 and MoCul4.** (A) KOG classification of the differentially expressed genes in Δ*Mocul4* and Δ*Mokmt1*. The bar chart shows the percentage of DEGs assigned to each functional category. (B) qPCR results for *MoMAT1-1* (*MGG_02979*) and *MoMAT A1-1* *(MGG_02978*), located in heterochromatin regions, showing significant upregulation in the mutants compared to wild type (n = 3 biological replicates per strain). Gene expression levels were normalized to the *β-TUBULIN* and *H3* reference genes and were shown relative to the wild type. Data are presented as means ± SD. Statistical significance was determined using two-tailed Student’s t-test (**P* < 0.05 compared to the wild type). (C) Heatmap analysis of β-glucanase genes showing ≥ 2-fold up- or down-regulation in Δ*Mocul4* and Δ*Mokmt1*.

**Figure 7. Schematic diagram of MoCul4 and MoKmt1 functions in *Magnaporthe oryzae*.**

**Supplementary Figure S1. Phylogenetic analysis of Cul4 homologs in** ***Magnaporthe oryzae*, other fungi, mammals and plants according to the BLAST result.** Abbreviations for fungal species followed by their NCBI accession numbers are as follows: *Fusarium graminearum* (Fg), *Fusarium oxysporum* (Fo), *Colletotrichum gloeosporioides* (Cg), *Neurospora crassa* (Nc), *Botrytis cinerea* (Nc), *Sclerotinia sclerotiorum* (Ss), *Magnaporthe oryzae* (Mo), *Parastagonospora nodorum* (Pn), *Zymoseptoria tritici* (Zt), *Penicillium chrysogenum* (Pc), *Aspergillus fumigatus* (Af), *Aspergillus nidulans* (An), *Schizosaccharomyces pombe* (Sp), *Cryptococcus neoformans* (Cn), *Mus musculus* (Mm), *Rhizopus delemar* (Rd), *Zea mays* (Zm), *Oryza sativa* (Os), *Arabidopsis thaliana* (At), *Drosophila melanogaster* (Dm), *Homo sapiens* (Hs), and *Saccharomyces cerevisiae* (Sc).

**Supplementary Figure S2. The sequence alignment of Cul4 homologs from *Neurospora crassa* (NcCul4), *Magnaporthe oryzae* (MoCul4), *Drosophila melanogaster* (DmCul4), *Homo sapiens* (Cullin-4B), *Caenorhabditis elegans* (CeCul4), and *Schizosaccharomyces pombe* (SpCul4).**

**Supplementary Figure S3. Verification of the Δ*Mokmt1* and Δ*Mocul4* mutants and the complement strain by PCR.** (A) Verification of the Δ*Mokmt1* and Δ*Mocul4* mutants by double PCR. Band I: a ~500-bp fragment of the *β-TUBULIN* gene (internal control). Band II: a ~250-bp fragment from coding sequence (CDS) of *MoCUL4* or *MoKMT1* (indicating successful knockout). Band III: a ~2-kb fragment amplified by primers flanking the outsider genome region upstream and the SUR resistance gene cassette (confirming correct gene replacement). (B) RT-PCR analysis confirming the restoration of *MoCUL4* or *MoKMT1* expression in the respective complement strains.

**Supplementary Figure S4. Expression of MoKmt1 at the transcriptional and protein levels in the wild type and Δ*Mocul4*.** (A) Relative expression levels of *MoKMT1* determined by qRT‑PCR. Gene expression levels were normalized to the *β-TUBULIN* and *H3* reference genes and were shown relative to the wild type (n = 3 biological replicates per strain). Data are presented as means ± SD. Statistical significance was determined using two-tailed Student’s t-test (**P* < 0.05 compared to the wild type). (B) Western blot analysis of GFP-MoKmt1 protein levels driven by its native promoter in wild type and Δ*Mocul4*.

**Supplementary Figure S5. Expression patterns of glucanase and transcription factor genes in Δ*Mokmt1* and Δ*Mocul4* in mycelia, 4-hpi appressoria, and invasive hyphae.** Gene expression levels were normalized to the *β-TUBULIN* and *H3* reference genes and were shown relative to the wild type. Statistical significance was determined using two-tailed Student’s t-test (**P* < 0.05 compared to the wild type).
